# Supplementary material for: Nationality dominates gender in decision-making in the Dictator and Prisoner’s Dilemma Games
Source: PLoS One. 2021 Jan 13;16(1):e0244568. doi: 10.1371/journal.pone.0244568 (PMC7806153; doi:10.1371/journal.pone.0244568)
Supplement: S1 File — (ZIP) [file pone.0244568.s001.zip › S5_File.docx]

**S5 Table.** **Models for Additivity (H1) across the Dictator Game, Prisoner’s Dilemma Decisions, and Prisoner’s Dilemma Beliefs.** Covariates are age, salary, education and wave of data collection.

|  | **DG** | **DG with Covariates** | **PD Decisions** | **PD Decisions with Covariates** | **PD Beliefs** | **PD Beliefs with Covariates** |
| --- | --- | --- | --- | --- | --- | --- |
| (Intercept) | 3.14 (0.19)^***^ | 2.90 (0.35)^***^ | -1.91 (0.54)^***^ | -1.54 (0.97) | -1.11 (0.38)^**^ | -1.24 (0.68) |
| Nationality group status | 0.39 (0.06)^***^ | 0.41 (0.06)^***^ | -0.73 (0.22)^**^ | -0.74 (0.23)^**^ | -0.94 (0.20)^***^ | -0.92 (0.21)^***^ |
| Gender group status | -0.06 (0.06) | -0.05 (0.06) | -0.14 (0.22) | -0.13 (0.23) | -0.27 (0.20) | -0.21 (0.20) |
| Participant nationality | -0.20 (0.19) | -0.36 (0.24) | 2.11 (0.53)^***^ | 1.22 (0.64) | 1.63 (0.37)^***^ | 1.34 (0.45)^**^ |
| Participant gender | -0.23 (0.19) | -0.19 (0.19) | 0.34 (0.49) | 0.29 (0.53) | -0.17 (0.35) | -0.20 (0.37) |
| Nationality group status X Gender group status | -0.00 (0.08) | -0.01 (0.09) | -0.01 (0.31) | 0.02 (0.33) | 0.34 (0.28) | 0.23 (0.29) |
| Age (18-34) |  | -0.65 (0.52) |  | -0.50 (1.44) |  | -0.85 (1.00) |
| Age (35-64) |  | -0.81 (0.32)^*^ |  | 0.81 (0.89) |  | 0.33 (0.62) |
| Salary ($0 - $25,000) |  | 0.01 (0.17) |  | 1.44 (0.48)^**^ |  | 0.39 (0.32) |
| Salary ($25,001 - $50,000) |  | -0.17 (0.18) |  | 0.26 (0.47) |  | -0.15 (0.33) |
| Education (Less than 4-year college degree) |  | -0.06 (0.21) |  | -1.29 (0.58)^*^ |  | -0.31 (0.40) |
| Education (4-year college degree) |  | -0.01 (0.16) |  | -0.62 (0.43) |  | -0.29 (0.30) |
| Wave 2018 Summer |  | 0.03 (0.24) |  | 0.28 (0.66) |  | 0.33 (0.46) |
| Wave 2018 Winter |  | -0.14 (0.23) |  | -0.65 (0.63) |  | -0.47 (0.44) |
| AIC | 6640.73 | 6314.95 | 1808.26 | 1703.97 | 2013.01 | 1907.25 |
| BIC | 6685.25 | 6403.18 | 1847.22 | 1786.69 | 2051.96 | 1989.97 |
| Log Likelihood | -3312.36 | -3141.47 | -897.13 | -836.99 | -999.51 | -938.63 |
| Num. obs. | 1931 | 1835 | 1932 | 1835 | 1929 | 1835 |
| Num. groups: subject | 483 | 459 | 483 | 459 | 483 | 459 |
| Var: subject (Intercept) | 3.90 | 3.85 | 17.73 | 18.51 | 9.64 | 9.67 |
| Var: Residual | 0.86 | 0.85 |  |  |  |  |

*Note.* AIC = Akaike information criterion; BIC = Bayesian information criterion; Num. obs. = number of observations; Var: subject variance associated with participant id (random intercept term). Baselines are as follows: ingroup for both nationality and gender group statuses; US American for participant nationality; male for participant gender; ‘Greater than 65’ for age; ‘More than $50,000’ for salary; ‘More than 4-year college degree’ for education; and ‘2017’ for wave. ^***^p < .001, ^**^p < .01, ^*^p < .05.


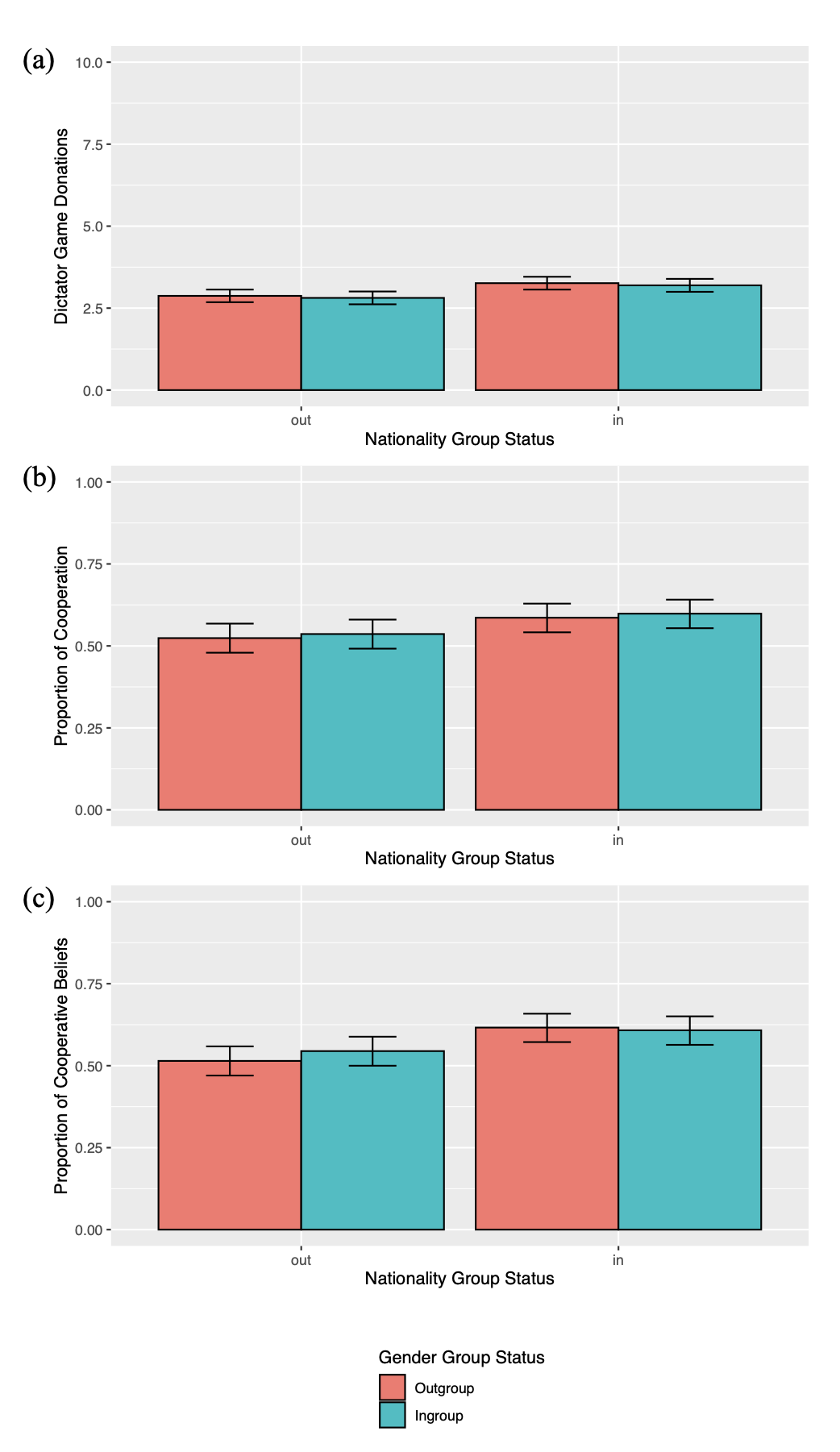


**S5 Figure.** Nationality and gender were not additive; instead, shared nationality increased cooperation. (a) Donations in the Dictator Game, (b) the proportions of decisions to cooperate in the Prisoner’s Dilemma and (c) the proportions of beliefs that partners will cooperate in the Prisoner’s Dilemma by whether nationality (x-axis), and/or gender (legend) are shared between participant and partner. All error bars represent 95% confidence intervals.
