# Supplementary material for: Nationality dominates gender in decision-making in the Dictator and Prisoner’s Dilemma Games
Source: PLoS One. 2021 Jan 13;16(1):e0244568. doi: 10.1371/journal.pone.0244568 (PMC7806153; doi:10.1371/journal.pone.0244568)
Supplement: S1 File — (ZIP) [file pone.0244568.s001.zip › S2_Appendix.docx]

**S2 Appendix. Recruitment & data cleaning**

For summary of the following information, please see S2 Table below.

We aimed to recruit 100 participants for each of the four social categories (i.e., Indian female, Indian male, US American female, US American male). Initially, 200 US American and 200 Indian participants were recruited through Amazon’s Mechanical Turk in September 2017. Our participants first completed our main survey including the identification questions and economic games through Qualtrics, then were directed to complete the AMP procedure, and finally returned to a demographic survey on Qualtrics. From the first 400 recruited participants, we had 572 data points in our main survey. We then removed participants who had duplicate MTurk ID’s (*N* = 31), participants who were not paid through MTurk (*N* = 148), and participants who did not belong to one of the four social categories (*N* = 5), resulting in 388 valid participants. However, these participants were highly imbalanced in terms of social category membership, with 53 Indian females, 128 Indian males, 85 US American females, and 122 US American males.

In an attempt to balance the cell sizes we recruited 150 Indians, 80 US Americans, and an additional 60 Indian females. Following the cleaning procedure identical to the one described above yielded a data set of 82 Indian females, 110 Indian males, 36 US American females, and 48 US American males. Through these two rounds we had collected 664 valid data points with the following breakdown: 135 Indian females, 238 Indian males, 121 US American females, and 170 US American males.

However, the surveys used for the first and second rounds of data collection differed in their comprehension checks for the Dictator Game and Prisoner’s Dilemma. Specifically, in the first iteration, participants saw instructions for both the game, and then had two chances to respond to comprehension check questions. After two failed attempts, participants were kicked out of the survey. We noticed that this resulted in a very high loss in data. Hence in the second iteration, participants who failed the first two attempts were given a third chance, before which they were reminded of the instructions for both games. Even when all three attempts were failed, participants were still able to complete the survey.

When looked at in isolation, our second round of data collection (*N* = 276; 82 Indian females, 110 Indian males, 36 US American females, and 48 US American males) needed more recruitment to reach the initial goal of 100 per cell.

Then, a research assistant blind to all hypotheses looked at just the second data set (*N* = 276) to check to see if the cell sizes were sufficient and comparable in the subset of participants who successfully passed our comprehension checks. Based on the comprehension check exclusions, a dropout rate was calculated for each social category. There were 71 successes for Indian females, but we needed 37 participants to get to 100 due to the 25.4% dropout rate; 94 successes for Indian males, but we needed 8 participants to get to 100 due to the 31.9% dropout rate; 33 successes for US American females, but we needed 76 participants to get to 100 due to the 12.1% dropout rate; 40 successes for US American males, but we needed 78 participants to get to 100 due to the 30% dropout rate. The numbers of needed participants were rounded up to the nearest whole number. In the final top up, we recruited 40 Indian females, 10 Indian males, 80 US American females, and 80 US American males. 31 Indian females, 11 Indian males, 77 US American females, and 77 US American males.

In total, the three rounds of recruitment and cleaning added up to 860 participants. However, out of the 860 who completed the first part of the survey, 666 participants completed the AMP and second survey as well. These 666 participants comprise our final data set.

**S2 Table. Data collection & cleaning summary.** Table reads left to right, with right bottom corner reflecting the final sample.

| Batch | Requested from MTurk | Data points in main survey | Unique users (excluding duplicates) | Compensated users | Valid social category | Completed both surveys | Social category breakdown |
| --- | --- | --- | --- | --- | --- | --- | --- |
| September 2017 | 200 IND  200 USA | 572 | 541 | 393 | 388 | 206 | INDF 19  INDM 51  USF 53  USM 83 |
| February 2018 | 80 USA  150 IND  60 INDF | 615 | 442 | 279 | 276 | 266 | INDF 78  INDM 106  USF 35  USM 47 |
|  |  |  |  |  |  |  |  |
| July 2018 | INDF 40  INDM 10  USF 80  USM 80 | 472 | 336 | 199 | 196 | 194 | INDF 30  INDM 11  USF 76  USM 77 |
| **TOTALS** | USA 440  IND 460  = 900 | 1659 | 1314 | 870 | 860 | 666 | **INDF 127**  **INDM 168**  **USF 164**  **USM 207** |
