# Supplementary material for: Nationality dominates gender in decision-making in the Dictator and Prisoner’s Dilemma Games
Source: PLoS One. 2021 Jan 13;16(1):e0244568. doi: 10.1371/journal.pone.0244568 (PMC7806153; doi:10.1371/journal.pone.0244568)
Supplement: S1 File — (ZIP) [file pone.0244568.s001.zip › S7_File.docx]

**S7 Table 1. Models for explicit identification with nationality and gender, across our measures.**

|  | **Dictator Game** | | **Prisoner’s Dilemma Decisions** | | **Prisoner’s Dilemma Beliefs** | |
| --- | --- | --- | --- | --- | --- | --- |
|  | **Nationality** | **Gender** | **Nationality** | **Gender** | **Nationality** | **Gender** |
| (Intercept) | 4.61 (0.78)^***^ | 3.69 (0.69)^***^ | -5.34 (2.20)^*^ | -2.47 (1.83) | -1.74 (1.56) | -2.47 (1.83) |
| Participant nationality | -1.10 (0.82) | -0.28 (0.20) | 3.93 (2.29) | 2.24 (0.55)^***^ | 1.30 (1.64) | 2.24 (0.55)^***^ |
| Partner nationality | -0.09 (0.34) | -0.04 (0.04) | 2.07 (1.30) | -0.24 (0.16) | -0.41 (1.13) | -0.24 (0.16) |
| Nationality identification | -0.11 (0.09) |  | 0.26 (0.24) |  | -0.07 (0.17) |  |
| Participant gender | -0.28 (0.19) | 0.13 (0.78) | 0.44 (0.50) | -1.47 (2.09) | -0.14 (0.36) | -1.47 (2.09) |
| Partner gender | -0.10 (0.04)^*^ | -0.18 (0.29) | 0.55 (0.16)^***^ | 1.49 (1.13) | 0.63 (0.14)^***^ | 1.49 (1.13) |
| Gender identification |  | -0.04 (0.08) |  | 0.01 (0.21) |  | 0.01 (0.21) |
| **Participant nationality X**  **Partner nationality X**  **Nationality identification** | 0.11 (0.04)^**^ |  | 0.04 (0.16) |  | -0.26 (0.14) |  |
| **Participant gender X**  **Partner gender X**  **Gender identification** |  | -0.01 (0.04) |  | 0.06 (0.16) |  | 0.06 (0.16) |
| AIC | 6624.39 | 6739.66 | 1795.77 | 1819.37 | 1995.45 | 1819.37 |
| BIC | 6691.16 | 6806.45 | 1856.98 | 1880.60 | 2056.63 | 1880.60 |
| Log Likelihood | -3300.20 | -3357.83 | -886.89 | -898.68 | -986.72 | -898.68 |
| Num. obs. | 1927 | 1931 | 1928 | 1932 | 1925 | 1932 |
| Num. groups: subject | 482 | 483 | 482 | 483 | 482 | 483 |
| Var: subject (Intercept) | 3.87 | 3.88 | 18.59 | 17.50 | 10.19 | 17.50 |
| Var: Residual | 0.85 | 0.91 |  |  |  |  |

*Note.* AIC = Akaike information criterion; BIC = Bayesian information criterion; Num. obs. = number of observations; Var: subject variance associated with participant id (random intercept term). Baselines are as follows: US American for participant nationality; male for participant gender. ^***^p < .001, ^**^p < .01, ^*^p < .05.

**S7 Table 2. Models for AMP difference scores by nationality and gender, across our measures.**

|  | **Dictator Game** | | **Prisoner’s Dilemma Decisions** | | **Prisoner’s Dilemma Beliefs** | |
| --- | --- | --- | --- | --- | --- | --- |
|  | **Nationality** | **Gender** | **Nationality** | **Gender** | **Nationality** | **Gender** |
| (Intercept) | 3.55 (0.21)^***^ | 3.33 (0.20)^***^ | -3.28 (0.66)^***^ | -2.58 (0.57)^***^ | -2.21 (0.47)^***^ | -1.89 (0.41)*** |
| Participant nationality | -0.37 (0.21) | -0.25 (0.19) | 2.64 (0.65)^***^ | 2.31 (0.54)^***^ | 2.20 (0.48)^***^ | 1.78 (0.38)*** |
| Partner nationality | -0.31 (0.08)^***^ | -0.04 (0.04) | -0.02 (0.29) | -0.24 (0.16) | -0.07 (0.27) | -0.27 (0.14) |
| AMP nationality difference score | 0.87 (0.47) |  | -0.25 (1.30) |  | -3.37 (1.09)^**^ |  |
| Participant gender | -0.26 (0.19) | -0.18 (0.20) | 0.42 (0.53) | 0.34 (0.54) | -0.17 (0.39) | -0.12 (0.40) |
| Partner gender | -0.10 (0.04)^*^ | -0.03 (0.07) | 0.60 (0.17)^***^ | 0.72 (0.26)^**^ | 0.67 (0.15)^***^ | 0.75 (0.23)** |
| Participant nationality X Partner nationality | 0.51 (0.09)^***^ |  | -0.54 (0.37) |  | -0.65 (0.33) |  |
| Participant nationality X AMP nationality | -2.60 (0.64)^***^ |  | 5.31 (1.85)^**^ |  | 4.65 (1.44)^**^ |  |
| Partner nationality X AMP nationality | -1.77 (0.20)^***^ |  | 3.76 (0.89)^***^ |  | 6.06 (1.01)^***^ |  |
| **Participant nationality X Partner nationality X AMP nationality** | **2.66 (0.28)^***^** |  | **-6.96 (1.28)^***^** |  | **-8.01 (1.26)^***^** |  |
| AMP gender difference score |  | 2.69 (1.38) |  | -10.97 (3.98)^**^ |  | -6.55 (2.93)* |
| Participant gender X Partner gender |  | -0.01 (0.09) |  | -0.50 (0.34) |  | -0.48 (0.30) |
| Participant gender X AMP gender |  | -3.31 (1.57)^*^ |  | 10.24 (4.37)^*^ |  | 5.59 (3.23) |
| Partner gender X AMP gender |  | -0.57 (0.63) |  | 5.35 (2.87) |  | 4.40 (2.36) |
| **Participant gender X Partner gender X AMP gender** |  | **2.90 (0.71)^***^** |  | **-7.71 (3.08)^*^** |  | **-7.08 (2.60)**** |
| AIC | 6485.00 | 6621.84 | 1734.25 | 1783.66 | 1914.87 | 1985.33 |
| BIC | 6551.68 | 6688.53 | 1795.39 | 1844.80 | 1975.99 | 2046.45 |
| Log Likelihood | -3230.50 | -3298.92 | -856.12 | -880.83 | -946.44 | -981.66 |
| Num. obs. | 1915 | 1915 | 1916 | 1916 | 1913 | 1913 |
| Num. groups: subject | 479 | 479 | 479 | 479 | 479 | 479 |
| Var: subject (Intercept) | 3.82 | 3.85 | 20.05 | 17.67 | 11.57 | 9.55 |
| Var: Residual | 0.80 | 0.88 |  |  |  |  |

*Note.* AIC = Akaike information criterion; BIC = Bayesian information criterion; Num. obs. = number of observations; Var: subject variance associated with participant id (random intercept term). Baselines are as follows: US American for participant nationality; male for participant gender. ^***^p < .001, ^**^p < .01, ^*^p < .05.


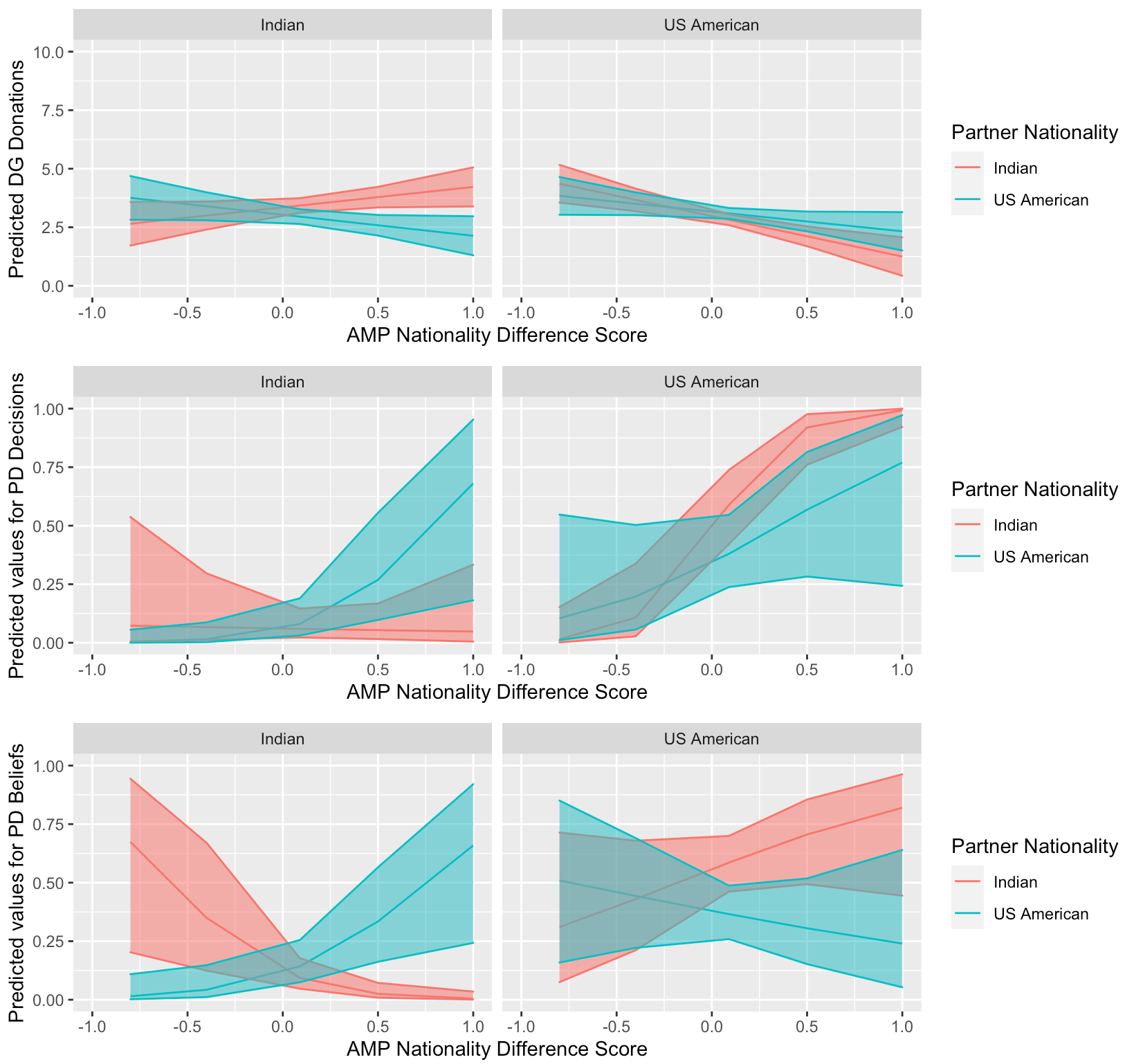


**S7 Fig 1.** Figures from models predicting donations in the Dictator Game (top row), the proportions of decisions to cooperate in the Prisoner’s Dilemma (second row) and the proportions of beliefs that partners will cooperate in the Prisoner’s Dilemma (third row), by AMP nationality difference scores (x-axis), and participant nationality (Indians = left column, US Americans = right column). Positive AMP scores indicate ingroup bias; negative scores indicate outgroup bias. Shaded areas represent 95% confidence regions.


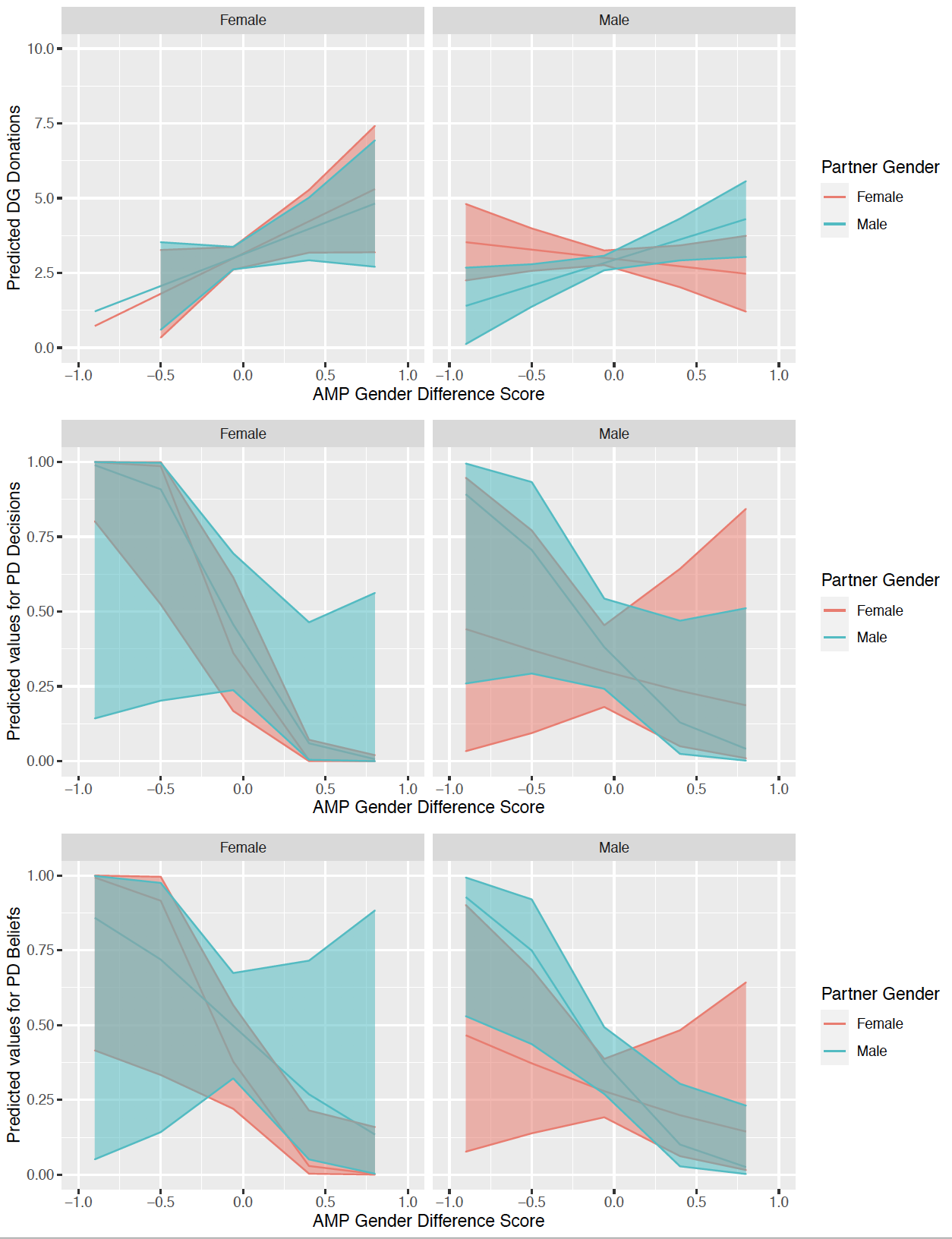


**S7 Fig 2.** Figures from models predicting donations in the Dictator Game (top row), the proportions of decisions to cooperate in the Prisoner’s Dilemma (second row) and the proportions of beliefs that partners will cooperate in the Prisoner’s Dilemma (third row), by AMP gender difference scores (x-axis), and participant gender (females = left column, males = right column). Positive AMP scores indicate ingroup bias; negative scores indicate outgroup bias. Shaded areas represent 95% confidence regions.
