# Supplementary material for: Nationality dominates gender in decision-making in the Dictator and Prisoner’s Dilemma Games
Source: PLoS One. 2021 Jan 13;16(1):e0244568. doi: 10.1371/journal.pone.0244568 (PMC7806153; doi:10.1371/journal.pone.0244568)
Supplement: S1 File — (ZIP) [file pone.0244568.s001.zip › S3_Table.docx]

**S3 Table. Demographic information of participants.** Presented in percentages of total (*N* = 479), broken down by social category membership.

|  | Overall | Indian Female | Indian Male | US American Female | US American Male |
| --- | --- | --- | --- | --- | --- |
| **Age** |  |  |  |  |  |
| 18-34 | 53.86 | 7.72 | 14.61 | 12.32 | 19.21 |
| 35-64 | 44.26 | 6.47 | 7.10 | 13.78 | 16.91 |
| 65+ | 1.46 | 0.21 | 0.00 | 0.42 | 0.84 |
| Did not report | 0.42 | 0.00 | 0.00 | 0.42 | 0.00 |
| **Salary** |  |  |  |  |  |
| $0 - $25,000 | 40.92 | 7.52 | 12.73 | 10.44 | 10.23 |
| $25,001 - $50,000 | 27.14 | 2.92 | 5.22 | 7.93 | 11.06 |
| More than $50,000 | 29.44 | 3.34 | 3.34 | 7.93 | 14.82 |
| Did not report | 2.51 | 0.63 | 0.42 | 0.63 | 0.84 |
| **Education** |  |  |  |  |  |
| Less than 4-year College Degree | 33.82 | 1.46 | 2.71 | 11.90 | 17.75 |
| 4-year College Degree | 46.56 | 7.31 | 13.15 | 11.90 | 14.20 |
| More than 4-year College Degree | 19.00 | 5.43 | 5.85 | 2.92 | 4.80 |
| Did not report | 0.63 | 0.21 | 0.00 | 0.21 | 0.21 |
