# Supplementary material for: Nationality dominates gender in decision-making in the Dictator and Prisoner’s Dilemma Games
Source: PLoS One. 2021 Jan 13;16(1):e0244568. doi: 10.1371/journal.pone.0244568 (PMC7806153; doi:10.1371/journal.pone.0244568)
Supplement: S1 File — (ZIP) [file pone.0244568.s001.zip › S6_File.docx]

**S6 Table. Models for Category Dominance (H2) with Covariates across our measures.** Covariates are age, salary, education and wave of data collection.

|  | **DG with Covariates** | **PD Decisions with Covariates** | **PD Beliefs with Covariates** |
| --- | --- | --- | --- |
| (Intercept) | 3.40 (0.35)^***^ | -2.75 (1.01)^**^ | -2.55 (0.71)^***^ |
| Participant nationality | -0.81 (0.24)^***^ | 1.96 (0.69)^**^ | 2.15 (0.50)^***^ |
| Partner nationality | -0.60 (0.07)^***^ | 0.54 (0.26)^*^ | 0.62 (0.24)^**^ |
| Participant gender | -0.15 (0.20) | 0.57 (0.57) | 0.03 (0.41) |
| Partner gender | -0.04 (0.07) | 0.90 (0.27)^***^ | 0.88 (0.24)^***^ |
| Participant nationality X  Partner nationality | 0.90 (0.09)^***^ | -1.42 (0.34)^***^ | -1.57 (0.31)^***^ |
| Participant gender X  Partner gender | -0.08 (0.09) | -0.52 (0.34) | -0.45 (0.30) |
| Age (18-34) | -0.65 (0.52) | -0.51 (1.49) | -0.88 (1.03) |
| Age (35-64) | -0.81 (0.32)^*^ | 0.84 (0.92) | 0.34 (0.64) |
| Salary ($0 - $25,000) | 0.01 (0.17) | 1.49 (0.49)^**^ | 0.40 (0.33) |
| Salary ($25,001 - $50,000) | -0.17 (0.18) | 0.27 (0.49) | -0.16 (0.34) |
| Education (Less than 4-year college degree) | -0.06 (0.21) | -1.33 (0.60)^*^ | -0.32 (0.41) |
| Education (4-year college degree) | -0.01 (0.16) | -0.65 (0.44) | -0.31 (0.31) |
| Wave 2018 Summer | 0.03 (0.24) | 0.29 (0.68) | 0.34 (0.47) |
| Wave 2018 Winter | -0.14 (0.23) | -0.67 (0.65) | -0.49 (0.45) |
| AIC | 6305.41 | 1690.77 | 1889.72 |
| BIC | 6399.16 | 1779.01 | 1977.96 |
| Log Likelihood | -3135.71 | -829.38 | -928.86 |
| Num. obs. | 1835 | 1835 | 1835 |
| Num. groups: subject | 459 | 459 | 459 |
| Var: subject (Intercept) | 3.85 | 19.55 | 10.23 |
| Var: Residual | 0.85 |  |  |

*Note.* AIC = Akaike information criterion; BIC = Bayesian information criterion; Num. obs. = number of observations; Var: subject variance associated with participant id (random intercept term). Baselines are as follows: US American for participant nationality; male for participant gender; ‘Greater than 65’ for age; ‘More than $50,000’ for salary; ‘More than 4-year college degree’ for education; and ‘2017’ for batch. ^***^p < .001, ^**^p < .01, ^*^p < .05.
