# Supplementary material for: Nationality dominates gender in decision-making in the Dictator and Prisoner’s Dilemma Games
Source: PLoS One. 2021 Jan 13;16(1):e0244568. doi: 10.1371/journal.pone.0244568 (PMC7806153; doi:10.1371/journal.pone.0244568)
Supplement: S1 File — (ZIP) [file pone.0244568.s001.zip › S4_Appendix.docx]

S4 Appendix. Surveys used in study.

Main Survey

Welcome!
 
Please enter your Amazon Mechanical Turk WorkerID. Please see below for where you can find your WorkerID. Your WorkerID starts with the letter A and has 12-14 letters or numbers. It is NOT your email address. If we do not have your correct WorkerID we will not be able to pay you. 
 
You will also be prompted in the next window to re-enter your ID for authentication purposes.  Thank you!

________________________________________________________________

**Boston College, Department of Psychology
 Informed Consent to be in study, Adult Online Informed Consent Form
 Researcher: Dr. Katherine McAuliffe**
 **Purpose:** We are conducting a research study to examine how people make decisions in social
 contexts.

 **Procedure:** Participation in this study involves completing a survey in which you will be asked to
 make decisions in different scenarios, to explain your attitudes, thoughts and opinions and/or to fill out some information about yourself. We anticipate that your involvement will require less than 15 minutes. To thank you for your participation, you will receive a small credit in your Amazon Mechanical Turk account.

 **Risks and Benefits:** There are no anticipated risks but there may be unknown risks, at this time. There are no costs associated with your participation in this study. Although this study will not benefit you personally, we hope that our results will add to our knowledge of social behavior and cognition.

 **Confidentiality:** All of your responses will be confidential. Only the researchers involved in this study and those responsible for research oversight at the university and government levels will have access to the information you provide. In any report we may publish, we will not include any information that will make it possible to identify you. Research records will be kept in a locked room and electronic records are stored on an encrypted server.

 **Voluntary Participation:** You were selected to be in this study because you are 18 years old or older and you clicked on our link on Amazon Mechanical Turk. Participation in this study is completely voluntary. You are free to decline to participate, to end participation at any time for any reason, or to refuse to answer any individual question without penalty or loss of compensation. Choosing not to participate will not affect your current or future relationship with Boston College.

 **Questions:** If you have any questions about this study, you may contact the research lab at
 bc.coop.questions@gmail.com. This research is funded by Professor McAuliffe’s research funds.

 If you have any questions about your rights as a research participant or concerns about the conduct of this study, you may contact the Office for Research Protections, 617-552-4778; irb@bc.edu.

 **Agreement to Participate:** In order to maintain anonymity, we will not ask you to sign a form to
 agree to participate. Instead agreement to participate will be indicated by your completing the
 following question and completing the following questionnaire and returning it to the experimenter. 

- I have read the above information and consent to participate (1)
- I have read the above information and do NOT consent to participate (2)

This HIT consists of three parts: (1) two decision making tasks with hypothetical partners, (2) a cognitive task and (3) a questionnaire. 
 
You will also be asked comprehension questions throughout the HIT. Please pay careful attention to the text in each question because **if you answer comprehension questions incorrectly, your HIT may be rejected**.
 
I identify my gender as:

- Male (1)
- Female (2)
- Transgender (3)
- Other (4) ________________________________________________
- Prefer not to say (5)

How important is your gender in describing who you are?

|  | Not important at all | Extremely important |
| --- | --- | --- |

|  | 0 | 1 | 2 | 3 | 4 | 5 | 6 | 7 | 8 | 9 | 10 |
| --- | --- | --- | --- | --- | --- | --- | --- | --- | --- | --- | --- |

| () | 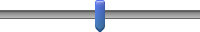 |
| --- | --- |

To what extent do you identify with your gender?

|  | Very little | A great deal |
| --- | --- | --- |

|  | 0 | 1 | 2 | 3 | 4 | 5 | 6 | 7 | 8 | 9 | 10 |
| --- | --- | --- | --- | --- | --- | --- | --- | --- | --- | --- | --- |

| () | 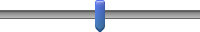 |
| --- | --- |

I identify my nationality as:

- US American (1)
- Indian (2)
- Other (3) ________________________________________________

How important is your nationality in describing who you are?

|  | Not important at all | Extremely important |
| --- | --- | --- |

|  | 0 | 1 | 2 | 3 | 4 | 5 | 6 | 7 | 8 | 9 | 10 |
| --- | --- | --- | --- | --- | --- | --- | --- | --- | --- | --- | --- |

| () | 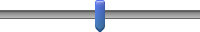 |
| --- | --- |

To what extent do you identify with your nationality?

|  | Very little | A great deal |
| --- | --- | --- |

|  | 0 | 1 | 2 | 3 | 4 | 5 | 6 | 7 | 8 | 9 | 10 |
| --- | --- | --- | --- | --- | --- | --- | --- | --- | --- | --- | --- |

| () | 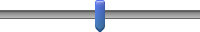 |
| --- | --- |

This task consists of four rounds. You are player A. Each round, you will be matched with a different Player B. **Please note that these are hypothetical decisions and partners**.
 
Each round you will be asked to imagine that you have been given a bonus of $10.00. You can choose how much of your bonus to transfer to Player B. Player B has to accept any amount of the bonus that you transferred to Player B.  For example:  if you transferred $0.00, then you will keep $10.00 and Player B will get $0.00.  If you transfer $2.00, then you will keep $8.00 and Player B will get $2.00. 
 
Before we get started, please answer a few quick questions to ensure you have understood the game:

In a given round of this game, if you choose to transfer $3.00 to Player B, how much would you keep?

- $2.00 (1)
- $7.00 (2)
- $3.00 (3)
- $5.00 (4)

Skip To: End of Block If In a given round of this game, if you choose to transfer $3.00 to Player B, how much would you keep? = $7.00

Display This Question:If In a given round of this game, if you choose to transfer $3.00 to Player B, how much would you keep? != $7.00

**The answer was incorrect. Please try again and answer the question with the correct answer.**

In a given round of this game, if you choose to transfer $3.00 to Player B, how much would you keep?

- $2.00 (1)
- $7.00 (2)
- $3.00 (3)
- $5.00 (4)

Skip To: End of Block If The answer was incorrect. Please try again and answer the question with the correct answer. In a... = $7.00

Display This Question:

If The answer was incorrect. Please try again and answer the question with the correct answer. In a... != $7.00

The answer was incorrect.  Please read the task instructions carefully and try again.
 

This task consists of four rounds. You are player A. Each round, you will be matched with a different Player B. Please note that these are hypothetical decisions and partners.
 
Each round you will be asked to imagine that you have been given a bonus of $10.00. You can choose how much of your bonus to transfer to Player B. Player B has to accept any amount of the bonus that you transferred to Player B.  For example:  if you transferred $0.00, then you will keep $10.00 and Player B will get $0.00.  If you transfer $2.00, then you will keep $8.00 and Player B will get $2.00. 
 
  In a given round of this game, **if you choose to transfer $3.00 to Player B, how much would you keep?**

- $2.00 (1)
- $3.00 (2)
- $7.00 (3)
- $5.00 (4)

Skip To: End of Block If The answer was incorrect.  Please read the task instructions carefully and try again.   This task... != $7.00

Imagine you are paired with the following Player B:


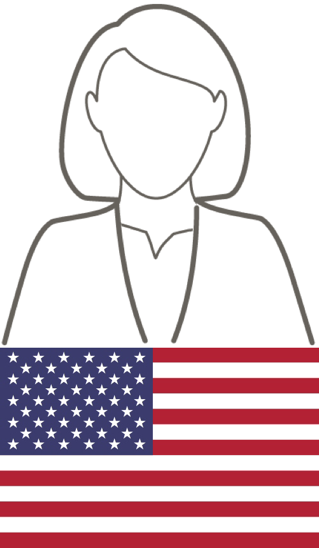


Please select how much you would send to Player B

- Transfer $0.00 to Player B (keep $10.00 for myself) (12)
- Transfer $1.00 to Player B (keep $9.00 for myself) (2)
- Transfer $2.00 to Player B (keep $8.00 for myself) (3)
- Transfer $3.00 to Player B (keep $7.00 for myself) (4)
- Transfer $4.00 to Player B (keep $6.00 for myself) (5)
- Transfer $5.00 to Player B (keep $5.00 for myself) (6)
- Transfer $6.00 to Player B (keep $4.00 for myself) (7)
- Transfer $7.00 to Player B (keep $3.00 for myself) (8)
- Transfer $8.00 to Player B (keep $2.00 for myself) (9)
- Transfer $9.00 to Player B (keep $1.00 for myself) (10)
- Transfer $10.00 to Player B (keep $0.00 for myself) (11)

Imagine you are paired with the following Player B:


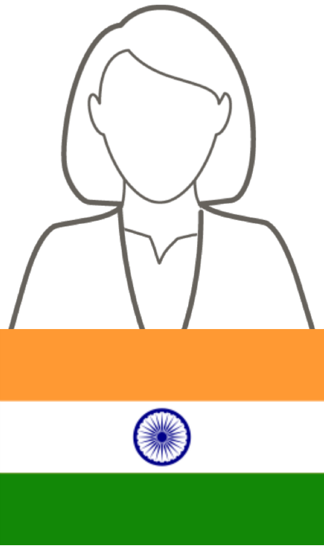


Please select how much you would send to Player B

- Transfer $0.00 to Player B (keep $10.00 for myself) (12)
- Transfer $1.00 to Player B (keep $9.00 for myself) (2)
- Transfer $2.00 to Player B (keep $8.00 for myself) (3)
- Transfer $3.00 to Player B (keep $7.00 for myself) (4)
- Transfer $4.00 to Player B (keep $6.00 for myself) (5)
- Transfer $5.00 to Player B (keep $5.00 for myself) (6)
- Transfer $6.00 to Player B (keep $4.00 for myself) (7)
- Transfer $7.00 to Player B (keep $3.00 for myself) (8)
- Transfer $8.00 to Player B (keep $2.00 for myself) (9)
- Transfer $9.00 to Player B (keep $1.00 for myself) (10)
- Transfer $10.00 to Player B (keep $0.00 for myself) (11)

Imagine you are paired with the following Player B:


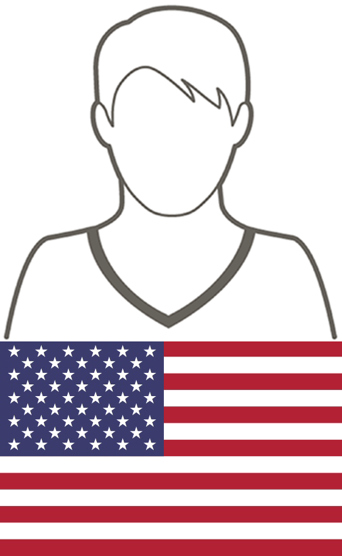


Please select how much you would send to Player B

- Transfer $0.00 to Player B (keep $10.00 for myself) (12)
- Transfer $1.00 to Player B (keep $9.00 for myself) (2)
- Transfer $2.00 to Player B (keep $8.00 for myself) (3)
- Transfer $3.00 to Player B (keep $7.00 for myself) (4)
- Transfer $4.00 to Player B (keep $6.00 for myself) (5)
- Transfer $5.00 to Player B (keep $5.00 for myself) (6)
- Transfer $6.00 to Player B (keep $4.00 for myself) (7)
- Transfer $7.00 to Player B (keep $3.00 for myself) (8)
- Transfer $8.00 to Player B (keep $2.00 for myself) (9)
- Transfer $9.00 to Player B (keep $1.00 for myself) (10)
- Transfer $10.00 to Player B (keep $0.00 for myself) (11)

Imagine you are paired with the following Player B:


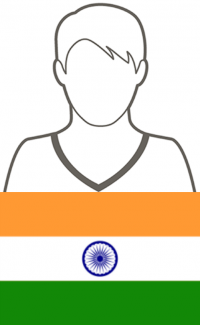


Please select how much you would send to Player B

- Transfer $0.00 to Player B (keep $10.00 for myself) (12)
- Transfer $1.00 to Player B (keep $9.00 for myself) (2)
- Transfer $2.00 to Player B (keep $8.00 for myself) (3)
- Transfer $3.00 to Player B (keep $7.00 for myself) (4)
- Transfer $4.00 to Player B (keep $6.00 for myself) (5)
- Transfer $5.00 to Player B (keep $5.00 for myself) (6)
- Transfer $6.00 to Player B (keep $4.00 for myself) (7)
- Transfer $7.00 to Player B (keep $3.00 for myself) (8)
- Transfer $8.00 to Player B (keep $2.00 for myself) (9)
- Transfer $9.00 to Player B (keep $1.00 for myself) (10)
- Transfer $10.00 to Player B (keep $0.00 for myself) (11)

This task consists of four rounds. You are player A. Each round, you will be matched with a different Player B. **Please note that these are hypothetical decisions and partners**.
 
In each round, both you and your partner will start with $2.00. You both make a choice:

**Keep** your $2.00 or

**Transfer** your $2.00 to the other person, in which case they will get $4.00.
 
This means that:

- If you both choose to transfer, you each get $4.00.
- If neither of you transfers, you each get $2.00.

BUT

- If you transfer while your partner does not, you get $0.00 while your partner gets $6.00
- If you do not transfer while your partner does, you get $6.00 while your partner gets $0.00


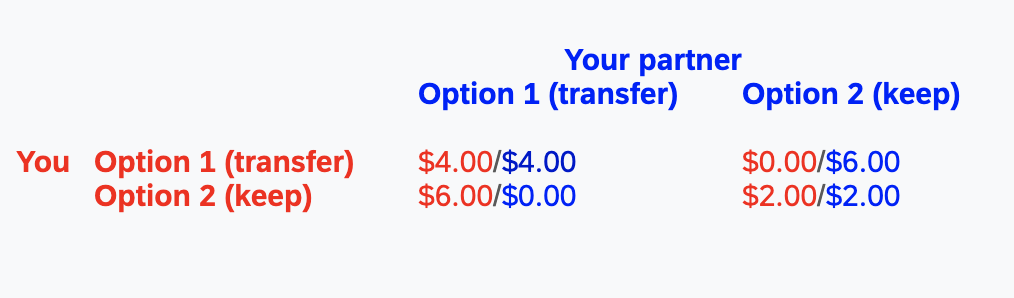


Before we get started, please answer a few quick questions to ensure you have understood the game: 

If you choose to keep $2.00 and your partner chooses to transfer $2.00 to you, what will your payoff be?

- $4.00 (1)
- $6.00 (2)
- $2.00 (3)
- $0.00 (4)

Skip To: End of Block If If you choose to keep $2.00 and your partner chooses to transfer $2.00 to you, what will your pay... = $6.00

Display This Question:

If If you choose to keep $2.00 and your partner chooses to transfer $2.00 to you, what will your pay... != $6.00

| 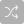 |
| --- |

**The answer was incorrect. Please try again and answer the question with the  correct answer.**
  If you choose to keep $2.00 and your partner chooses to transfer $2.00 to you, what will your payoff be?

- $4.00 (1)
- $6.00 (2)
- $2.00 (3)
- $0.00 (4)

Skip To: End of Block If The answer was incorrect. Please try again and answer the question with the  correct answer.    I... = $6.00

Display This Question:

If The answer was incorrect. Please try again and answer the question with the  correct answer.    I... != $6.00

| 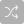 |
| --- |

The answer was incorrect.  Please read the task instructions carefully and try again.

  You are player A. Each round, you will be matched with a different Player B. **Please note that these are hypothetical decisions and partners**. In each round, both you and your partner will start with $2.00. You both make a choice:

**Keep**your $2.00 or

**Transfer**your $2.00 to the other person, in which case they will get $4.00.

This means that:

- If you both choose to transfer, you each get $4.00.
- If neither of you transfers, you each get $2.00.

BUT

- If you transfer while your partner does not, you get $0.00 while your partner gets $6.00
- If you do not transfer while your partner does, you get $6.00 while your partner gets $0.00    
        

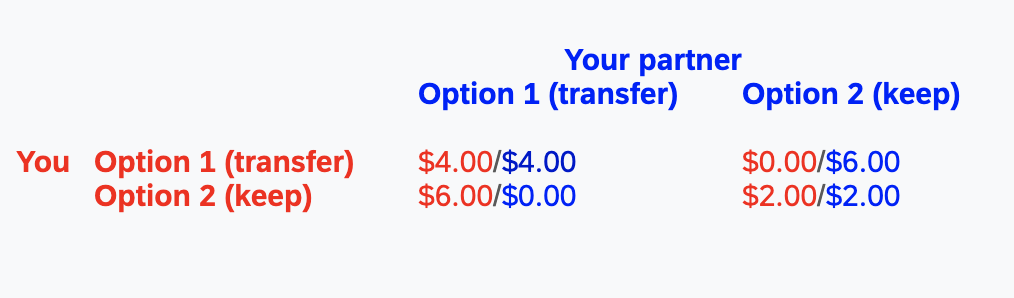


If you choose to keep $2.00 and your partner chooses to transfer $2.00 to you, what will your payoff be?

- $4.00 (1)
- $6.00 (2)
- $2.00 (3)
- $0.00 (5)

Skip To: End of Block If The answer was incorrect.  Please read the task instructions carefully and try again.   You are p... != $6.00

If you and your partner both choose to keep $2.00, what will your payoff be?

- $4.00 (1)
- $6.00 (2)
- $2.00 (3)
- $0.00 (4)

Skip To: End of Block If If you and your partner both choose to keep $2.00, what will your payoff be? = $2.00

Display This Question:

If If you and your partner both choose to keep $2.00, what will your payoff be? != $2.00

The answer was incorrect. Please try again and answer the question with the  correct answer. 
 
If you and your partner both choose to keep $2.00, what will your payoff be?

- $4.00 (1)
- $6.00 (2)
- $2.00 (3)
- $0.00 (4)

Skip To: End of Block If The answer was incorrect. Please try again and answer the question with the  correct answer.    I... = $2.00

Display This Question:

If The answer was incorrect. Please try again and answer the question with the  correct answer.    I... != $2.00

| 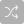 |
| --- |

The answer was incorrect.  Please read the task instructions carefully and try again.

  You are player A. Each round, you will be matched with a different Player B. **Please note that these are hypothetical decisions and partners**. In each round, both you and your partner will start with $2.00. You both make a choice:

**Keep**your $2.00 or

**Transfer**your $2.00 to the other person, in which case they will get $4.00.

This means that:

- If you both choose to transfer, you each get $4.00.
- If neither of you transfers, you each get $2.00.

BUT

- If you transfer while your partner does not, you get $0.00 while your partner gets $6.00
- If you do not transfer while your partner does, you get $6.00 while your partner gets $0.00    
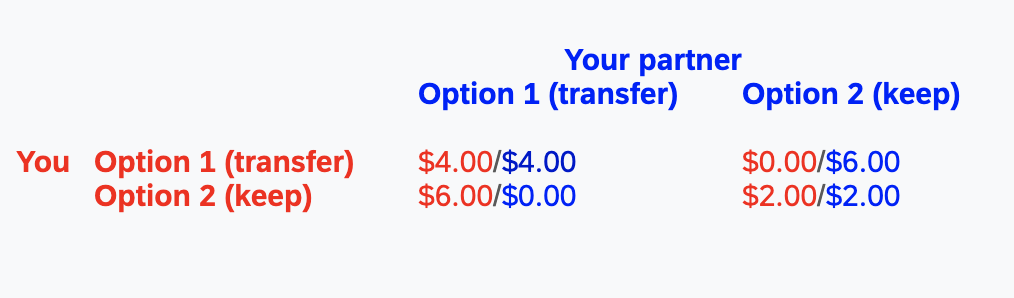


If you and your partner both choose to keep $2.00, what will your payoff be?

- $4.00 (1)
- $6.00 (2)
- $2.00 (3)
- $0.00 (4)

Skip To: End of Block If The answer was incorrect. Please try again and answer the question with the correct answer.     ... != $2.00

If you choose to transfer $2.00 to your partner and your partner chooses to keep $2.00, what will your payoff be?

- $4.00 (1)
- $6.00 (2)
- $2.00 (3)
- $0.00 (4)

Skip To: End of Block If If you choose to transfer $2.00 to your partner and your partner chooses to keep $2.00, what will... = $0.00

Display This Question:

If If you choose to transfer $2.00 to your partner and your partner chooses to keep $2.00, what will... != $0.00

**The answer was incorrect. Please try again and answer the question with the correct answer.**
  If you choose to transfer your $2.00 to your partner and your partner chooses to keep their $2.00, what will your payoff be?

- $4.00 (1)
- $6.00 (2)
- $2.00 (3)
- $0.00 (4)

Skip To: End of Block If The answer was incorrect. Please try again and answer the question with the  correct answer.    I... = $0.00

Display This Question:

If The answer was incorrect. Please try again and answer the question with the  correct answer.    I... != $0.00

The answer was incorrect.  Please read the task instructions carefully and try again.

  You are player A. Each round, you will be matched with a different Player B. **Please note that these are hypothetical decisions and partners**. In each round, both you and your partner will start with $2.00. You both make a choice:

**Keep**your $2.00 or

**Transfer**your $2.00 to the other person, in which case they will get $4.00.

This means that:

- If you both choose to transfer, you each get $4.00.
- If neither of you transfers, you each get $2.00.

BUT

- If you transfer while your partner does not, you get $0.00 while your partner gets $6.00
- If you do not transfer while your partner does, you get $6.00 while your partner gets $0.00    
        
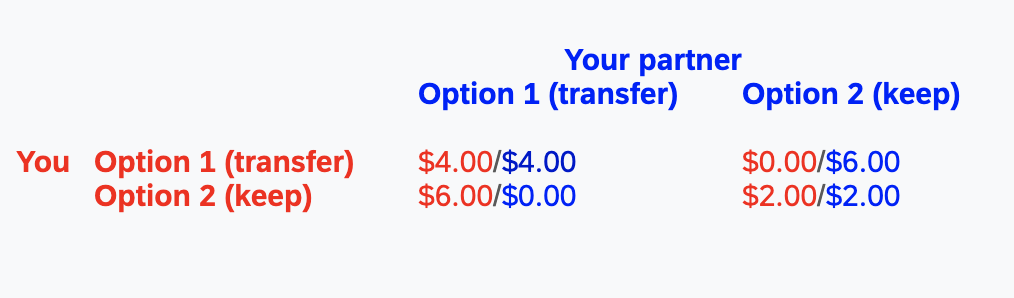


If you choose to transfer your $2.00 to your partner and your partner chooses to keep their $2.00, what will your payoff be?

- $4.00 (1)
- $6.00 (3)
- $2.00 (4)
- $0.00 (5)

Skip To: End of Block If The answer was incorrect. Please try again and answer the question with the correct answer.     ... != $0.00

If you choose to transfer $2.00 to your partner and they decide to transfer $2.00 to you, what will your payoff be?

- $4.00 (1)
- $6.00 (2)
- $2.00 (3)
- $0.00 (4)

Skip To: End of Block If If you choose to transfer $2.00 to your partner and they decide to transfer $2.00 to you, what wi... = $4.00

Display This Question:

If If you choose to transfer $2.00 to your partner and they decide to transfer $2.00 to you, what wi... != $4.00

Q68
**The answer was incorrect. Please try again and answer the question with the correct answer.**
  If you choose to transfer $2.00 to your partner and they decide to transfer $2.00 to you, what will your payoff be?

- $4.00 (1)
- $6.00 (2)
- $2.00 (3)
- $0.00 (4)

Skip To: End of Block If The answer was incorrect. Please try again and answer the question with the  correct answer.    I... = $4.00Display This Question:

If The answer was incorrect. Please try again and answer the question with the  correct answer.    I... != $4.00

| 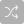 |
| --- |

The answer was incorrect.  Please read the task instructions carefully and try again.

  You are player A. Each round, you will be matched with a different Player B. **Please note that these are hypothetical decisions and partners**. In each round, both you and your partner will start with $2.00. You both make a choice:

**Keep**your $2.00 or

**Transfer**your $2.00 to the other person, in which case they will get $4.00.

This means that:

- If you both choose to transfer, you each get $4.00.
- If neither of you transfers, you each get $2.00.

BUT

- If you transfer while your partner does not, you get $0.00 while your partner gets $6.00
- If you do not transfer while your partner does, you get $6.00 while your partner gets $0.00    
        
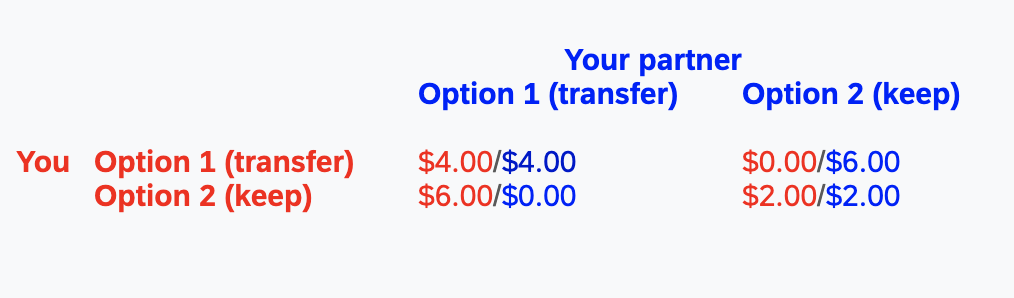


If you choose to transfer $2.00 to your partner and they decide to transfer $2.00 to you, what will your payoff be?

- $4.00 (1)
- $6.00 (2)
- $2.00 (3)
- $0.00 (4)

Skip To: End of Block If The answer was incorrect. Please try again and answer the question with the correct answer.     ... != $4.00

Imagine you are paired with the following Player B:


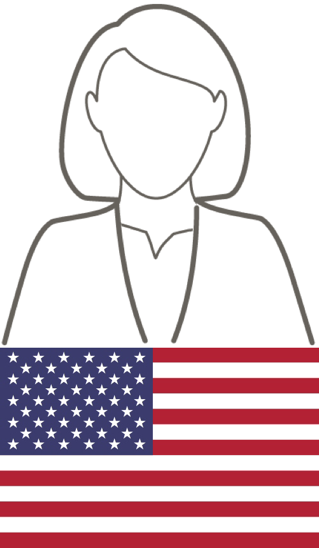


Which option would you choose?

- Transfer $2.00 to my partner (1)
- Keep $2.00 (2)

Which option do you think your partner would choose?

- Transfer $2.00 to you (1)
- Keep $2.00 (2)

Imagine you are paired with the following Player B:


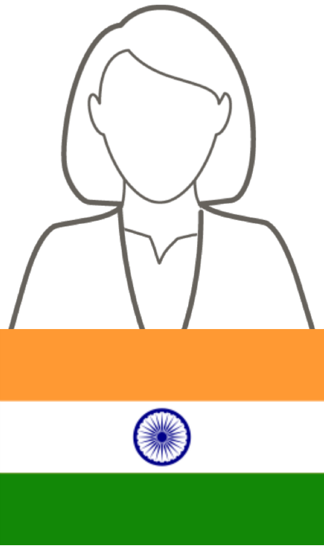


Which option would you choose?

- Transfer $2.00 to my partner (1)
- Keep $2.00 (2)

Which option do you think your partner would choose?

- Transfer $2.00 to you (1)
- Keep $2.00 (2)

Imagine you are paired with the following Player B:


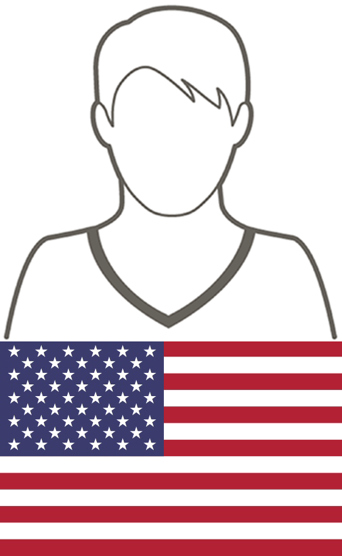


Which option would you choose?

- Transfer $2.00 to my partner (1)
- Keep $2.00 (2)

Which option do you think your partner would choose?

- Transfer $2.00 to you (1)
- Keep $2.00 (2)

Imagine you are paired with the following Player B:


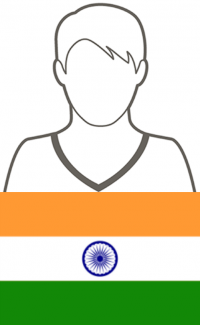


Which option would you choose?

- Transfer $2.00 to my partner (1)
- Keep $2.00 (2)

Which option do you think your partner would choose?

- Transfer $2.00 to you (1)
- Keep $2.00 (2)

Demographic Survey

*Completed after the Affect Misattribution Procedure.*

This is the third and final part of the study. In this part of the study, you will be asked to answer a series of questions. At the end of this section of the study, you will be given a completion code. tester

You just played a game in which we asked you to imagine that you had $10.00 and you were able to transfer some money to another player. You were then paired with four different hypothetical partners.

Who did you transfer the most money to? Please select all recipients of your highest transfer.

- American man (1)
- American woman (2)
- Indian man (3)
- Indian woman (4)
- I donated equally to all partners (5)

Who did you donate the least money to? Please select all recipients of your lowest transfer.

- American man (1)
- American woman (2)
- Indian man (3)
- Indian woman (4)
- I donated equally to all partners (5)

What influenced your decisions about how much to transfer when you were paired with different partners?

________________________________________________________________

You just played a game in which we asked you to imagine that both you and a partner had $2.00. You had the opportunity to transfer $2.00 to your partner or keep $2.00. Your partner had the same options. You were then paired with four different hypothetical partners.

To whom did you transfer $2.00? Please select all partners that apply.

- American woman (1)
- American man (2)
- Indian woman (3)
- Indian man (4)

Who did you expect would transfer $2.00 to you? Please select all partners that apply.

- American woman (1)
- American man (2)
- Indian woman (3)
- Indian man (4)

What influenced your decisions about whether or not to transfer $2.00 to different partners?

________________________________________________________________

What influenced your expectations about whether or not a partner would transfer $2.00 to you?

________________________________________________________________

Have you ever participated in a study similar to this one?

- Yes (1)
- No (3)

If yes, please briefly describe it:

________________________________________________________________

Do you have an idea of what we are studying? Please explain below:

________________________________________________________________

Do you speak Mandarin

- Yes (5)
- No (6)

Do you speak Cantonese

- Yes (1)
- No (2)

You saw a total of 120 pictographs in the cognitive task. How many did you recognize? Enter 0 if you did not recognize any.

________________________________________________________________

My annual salary (including commissions and bonuses) in US dollars is:

- $0 - $25,000 (1)
- $25,001 - $50,000 (2)
- $50,001 - $75,000 (3)
- $75,001 - $100,000 (4)
- $100,001 - $125,000 (5)
- $125,001 - $150,000 (6)
- $150,001 - $175,000 (7)
- $175,001 - $200,000 (8)
- $200,001+ (9)
- I would rather not say (10)

The highest level of education that I have completed is:

- Less than High School (1)
- High School / GED (2)
- Some College (3)
- 2-year College Degree (4)
- 4-year College Degree (5)
- Masters Degree (6)
- Doctoral Degree (7)
- Professional Degree (JD, MD) (8)
- I would rather not say (9)

My age is between:

- 18-24 (1)
- 25-34 (2)
- 35-54 (3)
- 55-64 (4)
- greater than 65 (5)
- I would rather not say (6)

**Survey Complete**

 You have now completed the survey. Please enter this completion code on Mechanical Turk to receive payment:

 **KF-A-${e://Field/idnum}**
